# Sarcoidosis: Solitary Pulmonary Mass Masquerading As Lung Cancer

**DOI:** 10.7759/cureus.81775

**Published:** 2025-04-05

**Authors:** Monika Ballabh, Srinath Ganesan, N Guru Prasad, T Raghupathy

**Affiliations:** 1 Department of General Surgery, Sree Balaji Medical College and Hospital, Chennai, IND; 2 Department of Surgical Oncology, Sree Balaji Medical College and Hospital, Chennai, IND

**Keywords:** chronic inflammation of skin, cutaneous, hilar lymphadenopathy, plaque sarcoidosis, pulmonary mass

## Abstract

Sarcoidosis is a multisystem inflammatory disease of unknown cause with a wide variety of presentations. It primarily involves the lungs and intrathoracic lymph nodes, as well as the eyes, skin, spleen, and liver. Very rarely, it can present as a solitary lung mass. We present a case of a 60-year-old female who presented with an erythematous plaque on her face associated with irritation. Initial imaging detected a well-defined lobulated mass in the right middle and lower lobes of the lung. A biopsy of the mass revealed non-caseating granulomas, suggestive of sarcoidosis.

## Introduction

Sarcoidosis is a systemic granulomatous disease of unknown etiology, characterized by the formation of non-caseating epithelioid granulomas in multiple organs. The lungs and intrathoracic lymph nodes are the most commonly affected sites, but the disease can also involve the skin, eyes, liver, spleen, heart, and nervous system. The exact pathogenesis of sarcoidosis remains unclear; however, it is believed to result from an exaggerated immune response to an unidentified antigen in genetically predisposed individuals. Environmental triggers, such as infectious agents (e.g., mycobacteria and propionibacteria), occupational exposures, and autoimmune dysregulation, have been implicated in disease onset [1]. The condition is more common among African Americans, with women having a higher lifetime incidence than men. First-degree relatives of African Americans have a threefold increased risk of developing the disease.

Pulmonary sarcoidosis classically presents with bilateral hilar lymphadenopathy, pulmonary infiltrates, and restrictive lung disease. However, atypical presentations, such as solitary pulmonary nodules or masses mimicking lung cancer, are rare but clinically significant [2]. The differentiation between pulmonary sarcoidosis and malignancy is challenging, as both can present with similar radiological features. In such cases, histopathological confirmation through biopsy is essential to establish the diagnosis.

Cutaneous manifestations of sarcoidosis occur in approximately 20%-35% of patients and can be the first indication of systemic involvement [3]. These include maculopapular eruptions, plaques, lupus pernio, and subcutaneous nodules. Skin biopsy revealing non-caseating granulomas is a key diagnostic feature.

Here, we present a rare case of sarcoidosis in a 60-year-old female who initially presented with erythematous cutaneous plaques, subsequently found to have a solitary pulmonary mass. This case underscores the importance of recognizing atypical presentations of sarcoidosis to avoid misdiagnosis and unnecessary interventions.

## Case presentation

A 60-year-old female presented with multiple reddish skin lesions over her face, chin, left back of the ear, and lower back of the trunk, which had been present for three months, and were increasing in size. The lesions were associated with itching. There was no history of any systemic involvement. General physical and systemic examinations were normal. The serum angiotensin-converting enzyme (ACE) titer was 66 U/L. Serum calcium, liver function tests, renal function tests, and abdominal ultrasonography were normal. Laboratory investigations are mentioned in Table 1.

**Table 1:** Laboratory test results with corresponding reference ranges ACE: angiotensin-converting enzyme; S: serum; ALP: alkaline phosphatase

Lab test	Value	Normal range
S.ACE	66 U/L	40-52 U/L
S.calcium	9 mg/dL	9-11 mg/dL
Total bilirubin	0.7 mg/dL	0.3-1.1 mg/dL
Direct bilirubin	0.2 mg/dL	0.0-0.2 mg/dL
ALP	62 IU/L	56-119 IU/L
S.urea	40 mg/dL	17-49 mg/dL
S.creatinine	0.9 mg/dL	0.9-1.3 mg/dL
S.uric acid	4.6 mg/dL	4.5-7.6 mg/dL

**Figure 1:**
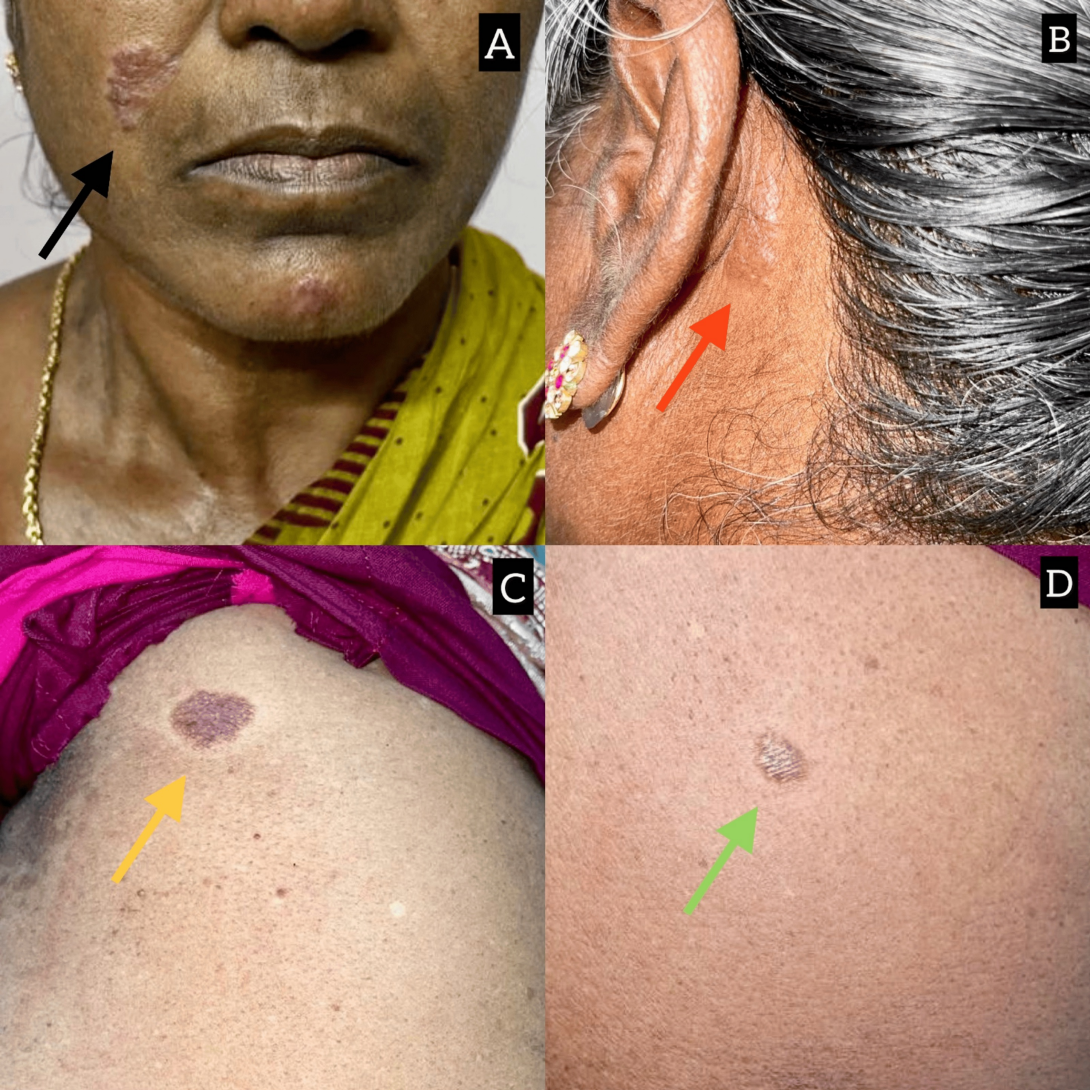
Gross images of the patient show multiple well-defined, erythematous, raised, reddish-brown, oval cutaneous lesions on the face, chin (black arrow), behind the left ear (red arrow), and on the lower back of the trunk (green and yellow arrows) A - Well-defined, erythematous, raised, reddish-brown, oval cutaneous lesions, on the face and chin B - Cutaneous lesion behind the left ear C - Hyperpigmented cutaneous lesion over the lower trunk D - Hyperpigmented cutaneous lesion over the lower trunk

Cutaneous examination revealed multiple well-defined, erythematous, raised, reddish-brown, oval cutaneous lesions, over the face, chin (Figure 1A), behind the left ear (Figure 1B), and lower back of the trunk (Figures 1C, 1D).

**Figure 2:**
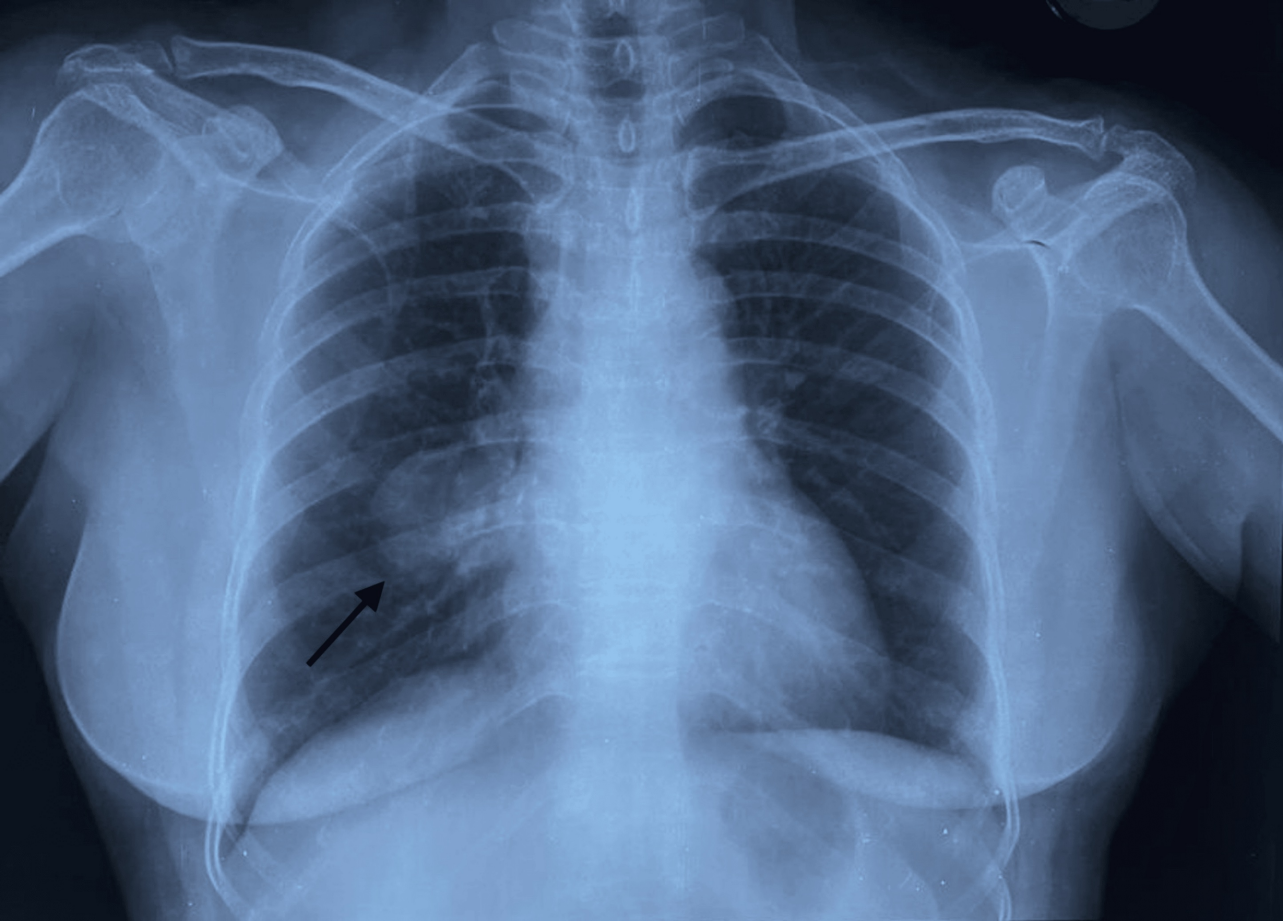
Chest radiograph of the patient showing a well-defined, smooth, radio-opaque mass in the lower zone of the right lung (black arrow)

A skin biopsy from the facial lesion revealed non-caseating epithelioid granulomas. Chest X-ray (CXR) revealed a smooth, well-defined, round, radio-opaque lesion (black arrow) noted in the lower zone of the right lung with no mass effect, and hilar vessels coursing through it, with a possibility of a solitary pulmonary nodule (Figure 2).

**Figure 3:**
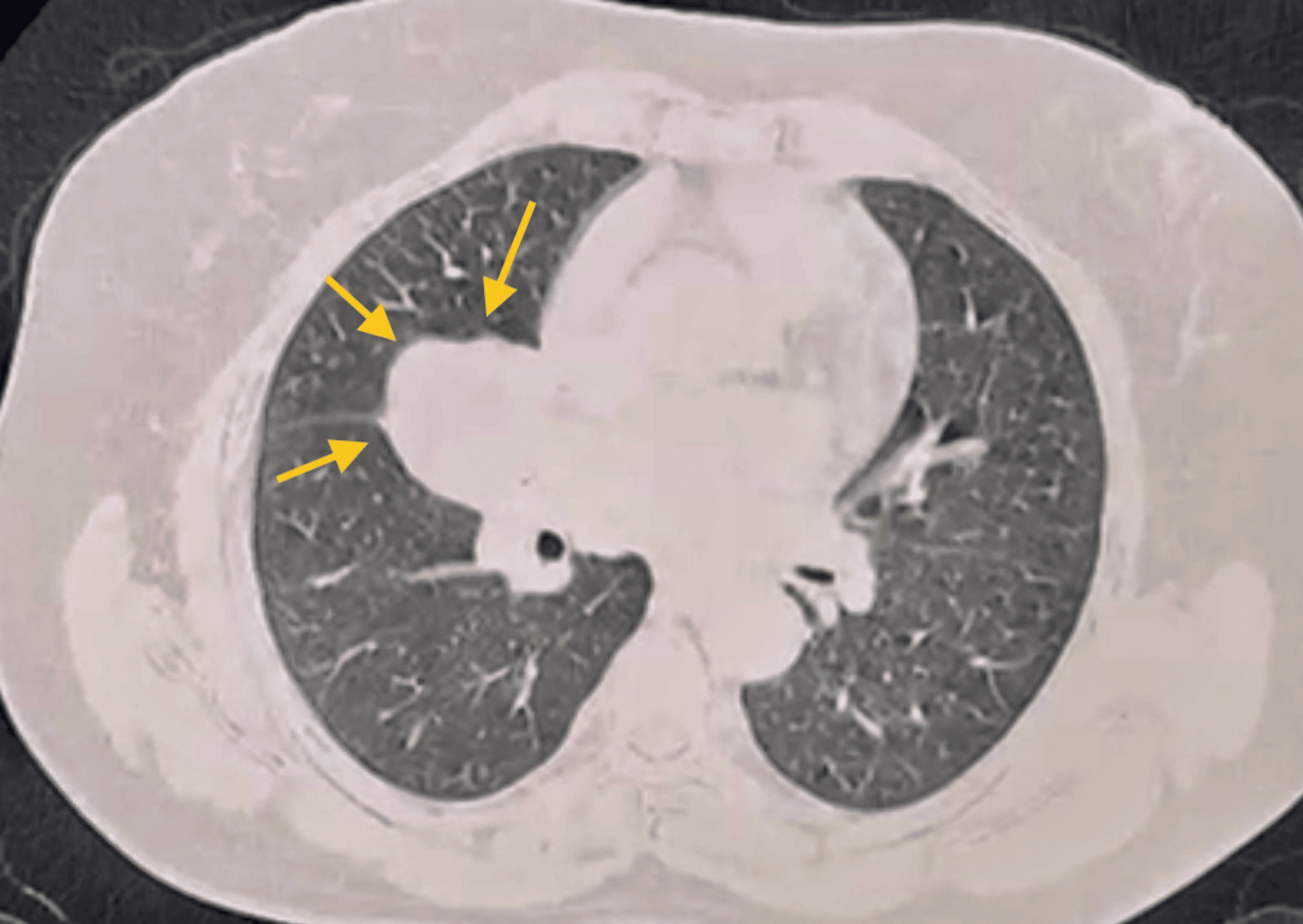
CT of the chest showed a large, well-defined, lobulated soft tissue density lesion (yellow arrows) in the right parahilar region, involving the right middle lobe and superior segment of the right lower lobe

CT of the chest showed a large, well-defined, lobulated soft tissue density lesion measuring approximately 4.0 cm × 4.2 cm × 3.7 cm in the right parahilar region, involving the right middle lobe and superior segment of the right lower lobe. The lesion was seen abutting the mediastinal pleura and the right middle lobe bronchi, with a few hilar lymph nodes present (Figure 3).

**Figure 4:**
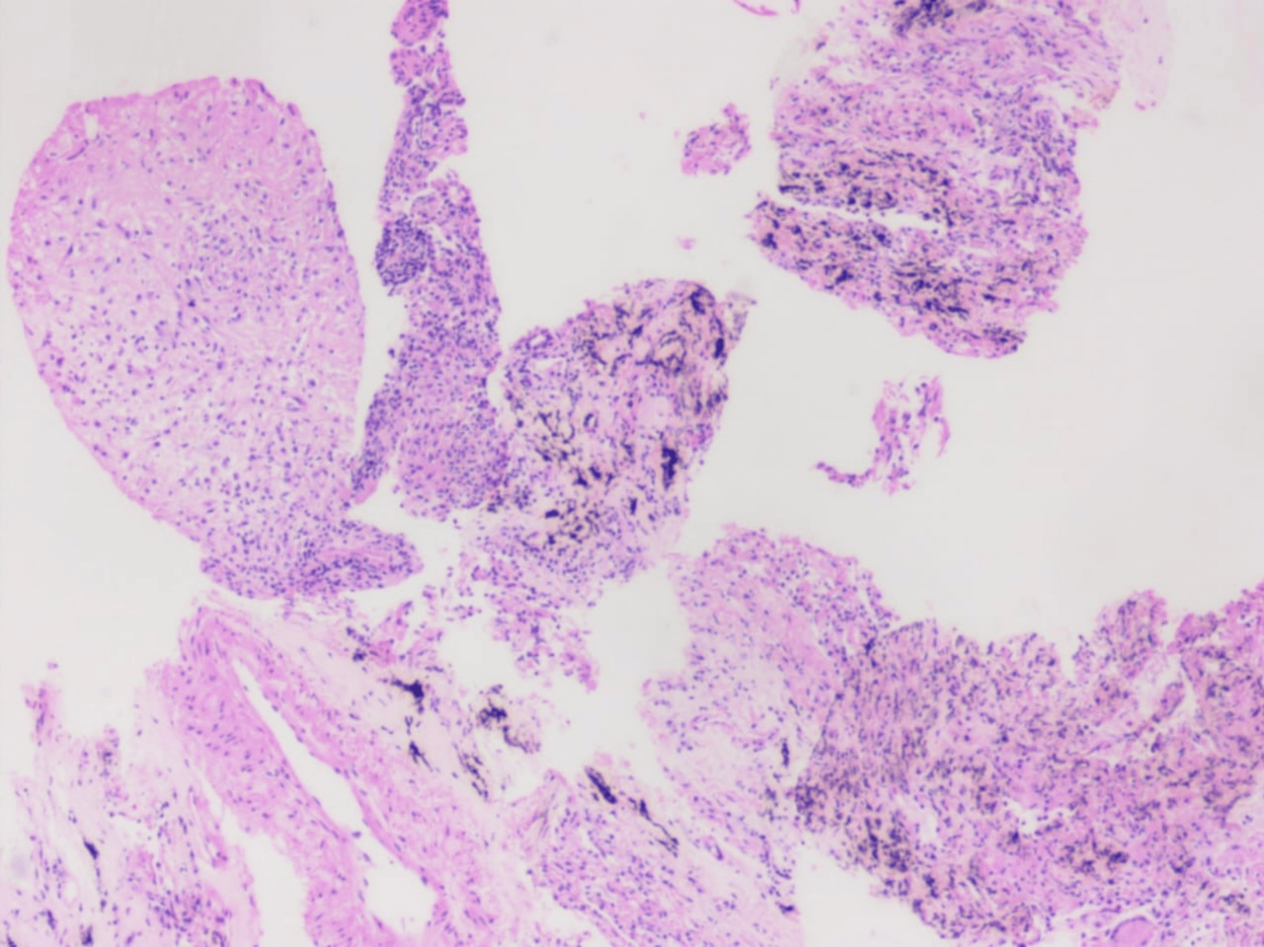
Histopathological image shows moderate infiltration of lymphocytes, focal anthrocotic pigment, and histiocytes forming ill-defined granulomas

A CT-guided biopsy of the right lung mass revealed a granulomatous lesion. Fite-Faraco staining and acid-fast bacilli (AFB) staining were negative for tuberculosis (TB). There was no evidence of malignancy (Figure 4).

## Discussion

Sarcoidosis is a multisystem inflammatory disease of unknown origin, believed to arise from a combination of genetic predisposition and environmental triggers. Although the precise cause remains unclear, the hallmark of sarcoidosis is the presence of non-caseating granulomas in affected organs, leading to inflammation and potential organ dysfunction [4]. Notably, sarcoidosis is distinct from malignancies and autoimmune disorders, despite sharing certain clinical characteristics with these conditions. The incidence of sarcoidosis varies significantly across racial and ethnic groups, with African Americans being disproportionately affected. The prevalence among African Americans is approximately 34 cases per 100,000 individuals, a rate notably higher than in other populations. Additionally, familial clustering of the disease is well-documented, with first-degree relatives of affected individuals exhibiting a 2.5-fold greater risk, reinforcing the role of genetic factors in its development. Women are diagnosed more frequently than men, with a male-to-female ratio of 1:2, suggesting a potential hormonal influence, though the underlying mechanisms remain uncertain [5,6].

The lungs are the most commonly affected organ in sarcoidosis, with pulmonary involvement frequently manifesting as hilar lymphadenopathy (enlargement of lymph nodes at the lung roots) and reticulonodular opacities on chest imaging. These radiographic features are characteristic of the disease but are not pathognomonic [7,8]. Bilateral hilar lymphadenopathy is a particularly notable finding, though it can also be observed in conditions such as infections (e.g., TB), lymphoma, or malignancies like lung cancer [9]. Distinguishing sarcoidosis from malignancy can be particularly challenging when the disease presents as a solitary pulmonary mass, as this appearance may closely resemble lung cancer.

Diagnosing pulmonary sarcoidosis in cases where a solitary mass is present requires a combination of imaging modalities, laboratory tests, and histopathological examination. CXR and high-resolution computed tomography (HRCT) are instrumental in detecting lung involvement, often revealing a well-defined, lobulated mass with or without hilar lymphadenopathy. Positron emission tomography-computed tomography (PET-CT) can demonstrate increased FDG uptake, which may mimic malignancy, necessitating histopathological confirmation [9]. Serum markers such as ACE and calcium levels may provide supportive evidence but lack diagnostic specificity. Tissue sampling is commonly performed using bronchoscopy with transbronchial lung biopsy (TBLB), CT-guided lung biopsy, or endobronchial ultrasound-guided fine-needle aspiration (EBUS-FNA). The defining histopathological feature is the presence of non-caseating granulomas, and additional special stains (e.g., AFB, Fite-Faraco) and cultures are required to exclude TB and fungal infections. Sarcoidosis staging is determined using chest radiographic findings, following the Scadding system, as summarized in Table 2 [10].

**Table 2:** Grading system for sarcoidosis CXR: chest X-ray

Stage	Radiographic findings	Prognosis
Stage 0	Normal CXR	Good
Stage I	Bilateral hilar lymphadenopathy	60%-80% spontaneous resolution
Stage II	Hilar adenopathy + parenchymal infiltrates	Variable prognosis may need treatment
Stage III	Parenchymal infiltrates without adenopathy	Progressive lung disease
Stage IV	Pulmonary fibrosis	Poor prognosis, irreversible damage

A concise tabular column of the various differential diagnoses is summarized as follows in Table 3.

**Table 3:** Various differential diagnosis of sarcoidosis HP: hypersensitivity pneumonitis; CBD: chronic beryllium disease; AFB: acid-fast bacilli; NTM: nontuberculous mycobacteria; TB: tuberculosis

Category	Differential diagnosis	Key features	Diagnostic differentiation
Infectious causes	Mycobacterial infections (TB and NTM)	Necrotizing or non-necrotizing granulomas and systemic symptoms	AFB staining, culture, and PCR
	Fungal infections (Histoplasmosis, Blastomycosis, and Coccidioidomycosis)	Pulmonary and systemic involvement and mediastinal lymphadenopathy	Fungal stains, serology, and culture
Immune-mediated	HP	History of antigen exposure and diffuse lung involvement	Bronchoalveolar lavage and biopsy showing small, poorly formed granulomas
	Granulomatosis with polyangiitis (Wegener's)	Vasculitis, necrotizing granulomas, and upper/lower respiratory involvement	ANCA testing and biopsy showing vessel involvement
Occupational/environmental	Pneumoconiosis (CBD)	History of exposure (beryllium, aluminum, and organic dust)	Beryllium lymphocyte proliferation test
Drug-induced	Drug-induced sarcoid-like reaction	Use of biologics, checkpoint inhibitors, and methotrexate	Resolves after discontinuation of the drug

Treatment is guided by the severity of symptoms and disease progression. Asymptomatic or minimally symptomatic cases may not require immediate intervention, as some solitary pulmonary masses regress spontaneously. For symptomatic or progressive cases, corticosteroids remain the first-line therapy, with prednisone 20-40 mg/day given for four to six weeks, followed by a gradual taper. Patients with recurrent or steroid-dependent disease may benefit from steroid-sparing immunosuppressants, such as methotrexate, azathioprine, or mycophenolate mofetil. In refractory cases, biological agents like TNF-alpha inhibitors (infliximab and adalimumab) may be considered [11]. Regular follow-up with imaging and pulmonary function tests is necessary to assess treatment response and prevent complications.

## Conclusions

Sarcoidosis is a multisystem inflammatory disease that most commonly affects the lungs, often presenting with bilateral hilar lymphadenopathy and reticulonodular opacities on imaging. Its presentation as a solitary pulmonary mass can closely mimic lung cancer, making differentiation challenging. Early diagnosis through biopsy and histopathological examination is crucial to avoid unnecessary invasive procedures and to ensure appropriate treatment, typically with corticosteroids. Understanding the clinical and radiological features of sarcoidosis, along with its diagnostic tools, is essential for effective management and to prevent potential complications.
